# Global demographic history of human populations inferred from whole mitochondrial genomes

**DOI:** 10.1098/rsos.180543

**Published:** 2018-08-22

**Authors:** Eleanor F. Miller, Andrea Manica, William Amos

**Affiliations:** Department of Zoology, University of Cambridge, Downing Street, Cambridge CB2 3EJ, UK

**Keywords:** demographic history, coalescent, mitochondrial DNA, Bayesian skyline plots

## Abstract

The Neolithic transition has led to marked increases in census population sizes across the world, as recorded by a rich archaeological record. However, previous attempts to detect such changes using genetic markers, especially mitochondrial DNA (mtDNA), have mostly been unsuccessful. We use complete mtDNA genomes from over 1700 individuals, from the 1000 Genomes Project Phase 3, to explore changes in populations sizes in five populations for each of four major geographical regions, using a sophisticated coalescent-based Bayesian method (extended Bayesian skyline plots) and mutation rates calibrated with ancient DNA. Despite the power and sophistication of our analysis, we fail to find size changes that correspond to the Neolithic transitions of the study populations. However, we do detect a number of size changes, which tend to be replicated in most populations within each region. These changes are mostly much older than the Neolithic transition and could reflect either population expansion or changes in population structure. Given the amount of migration and population mixing that occurred after these ancient signals were generated, we caution that modern populations will often carry ghost signals of demographic events that occurred far away from their current location.

## 1. Introduction

The Neolithic transition was associated with major cultural and societal changes, and a number of archaeological lines of evidence point to a rapid increase in census population size following the advent of food production and the associated sedentarism (reviewed in [1]). However, past attempts to detect such size changes using genetic markers have generally failed to find any signal attributable to the Neolithic transition [2–5]. When population changes were detected, these were generally dated to older times, leading to the suggestion that populations that later adopted agriculture might have started growing before the advent of food production [4].

A major difficulty in interpreting these results is that genetic dating of events is a very challenging endeavour, as mutation rates (which provide the molecular clock used to convert genetic changes into calendar years) come with high levels of uncertainty [6]. Over the last couple of years, the availability of ancient DNA, coupled with sophisticated tip-based calibration methods that use the age of ancient samples to estimate the rate at which differences between sequences accumulate, has greatly improved the accuracy of mutation rates, especially for mtDNA [7].

Here, we take advantage of the Phase 3 data of the 1000 Genomes Project, which now includes over 2500 individuals from several major continental regions [8]. We use extended Bayesian skyline plots (EBSPs) in BEAST to best reconstruct the changes in effective population size through time, and take advantage of leaf-calibrated mutation rates based on extensive data from ancient DNA [7,9–11]. While we build on several previous analyses that are conceptually similar e.g. [2,3,12], the current study includes a number of important technical advances that should improve our ability to detect any demographic signal of the Neolithic transition that might be present. Furthermore, compared to previous analyses based on the Phase 1 1000 Genomes data, we are now able to include five South Asian populations, sequenced as part of Phase 3.

## 2. Material and methods

### 2.1. Sampled populations

The Phase 3 sequence data from 20 populations, comprising five populations for each of the four main geographical regions of Europe, East Asia, South Asia and Africa, were downloaded from the 1000 Genomes Project website (www.1000genomes.org/data, [8]), including whole mitochondrial genome data for 1999 individuals. We decided not to analyse populations from the Americas due to the region's complex history of admixture [13,14].

The European populations were as follows: Finnish sampled in Finland (FIN); European Caucasians resident in Utah, USA (CEU); British in England and Scotland (GBR); an Iberian population from Spain (IBS) and Toscani from Italy (TSI). Representing East Asia were the Han Chinese in Beijing (CHB); Southern Han Chinese (CHS); Dai Chinese from Xishuangbanna, China (CDX); Kinh population from Ho Chi Minh City, Vietnam (KHV) and Japanese from Tokyo (JPT). The South Asian populations were Punjabi Indians from Lahore, Pakistan (PJL); Gujarati Indians in Houston, USA (GIH) as well as Indian Telugu sampled in the UK (ITU); Bengali from Bangladesh (BEB) and Sri Lankan Tamil from the UK (STU). Finally, in Africa, we chose a population from the Western Division within The Gambia (GWD); Mende from Sierra Leone (MSL); the Yoruba from Nigeria (YRI); the Esan, also from Nigeria (ESN); as well as the Luhya from Webuye in Kenya (LWK). Full details of the populations and the original sampling and sequencing methods can be found on the 1000 Genomes Project website (www.1000genomes.org).

### 2.2. Data partitioning

Mutation rates of mtDNA vary among bases according to region, codon position and depending on whether the region is genic or non-genic [15]. We maximized the power of our analysis by accounting for these heterogeneities using the partitioning scheme developed by Rieux *et al*. [7], who used PartitionFinder [16] on a large panel of modern and ancient complete mtDNA genomes. Following their best model [7], the partitions, substitution model and rates were: the hypervariable segments 1 and 2 (HVS1 + HVS2) with a TN93 + I + G substitution model and a rate of 31.434 × 10^−8^ µ/Site/Year; rRNA and tRNA (r + tRNA) with TN93 + I + G and 1.007 × 10^−8^ µ/Site/Year; protein coding positions at 1st and 2nd codon (PC1 + PC2) with TN93 + I + G and 0.756 × 10^−8^ µ/Site/Year; and protein coding positions at the 3rd codon (PC3) with TN93 + G and 3.323 × 10^−8^ µ/Site/Year. See electronic supplementary material, table S1.

### 2.3. Data analysis

We analysed our mtDNA data with the extended Bayesian skyline plot (EBSP) method, a Bayesian, non-parametric technique for inferring past population size fluctuations from genetic data. Building on the previous Bayesian skyline plot (BSP) approach, EBSP uses a piecewise-linear model and Markov chain Monte Carlo (MCMC) methods to reconstruct a populations' demographic history [17] and is implemented in the software package BEAST v. 2.3.2 [11]. Alignments for each of the 20 populations were loaded separately into the Bayesian Evolutionary Analysis Utility tool (BEAUti v. 2.3.2) in NEXUS format. BEAUti is a graphical user interface that supports the creation of BEAST XML input files, enabling the user easily to set parameters and specific model criteria. Within BEAUti, a ‘Gamma Category Count’ of four was selected for partitions using +G models to allow for the inclusion of gamma rate heterogeneity. For partitions using +I models, the ‘Proportion Invariant’ was set to 0.1 and the ‘estimate’ box selected allowing the analysis to include a proportion of invariant sites. ‘Coalescent Extended Bayesian Skyline’ process was used and the ‘Population Model’ population factor set as 0.5 to account for the female only contribution to the *N*_e_ [17]. A linked, strict molecular clock and linked phylogenetic tree were used for all analyses. All other operator settings were left as default.

Each population was run separately with each run consisting of 100 million generations sampled every 10 000 steps and the first 10 million samples were discarded as burn-in [17]. To maximize comparability, the sample size used was 85 for all populations, equal to the smallest sample (MSL). Where more samples were available, 85 samples were selected at random. Each dataset was subject to two replicate runs to confirm repeatability. Runs were analysed using Tracer v. 1.6 and convergence was verified by plotting MCMC chain traces and ensuring that the effective sample sizes (ESS) of all relevant parameters exceeded 200. Independent runs were then combined using LogCombiner (v. 2.3.2) and again analysed using Tracer (v. 1.6) to determine that the same stationary distribution was sampled both times. Demographic reconstructions were then plotted in R (v. 3.2.3).

To confirm that 85 samples provide adequate data for accurate population reconstruction, we re-ran the analyses using all available samples for the population from each major region with the maximum samples. Run length was extended to 200 million generations to account for increased sample size, while burn-in remained at 10%. For the four major regions, these largest samples were: IBS in Europe (*n* = 107 samples); CHS in East Asia (*n* = 105 samples); GIH in South Asia (*n* = 103 samples); GWD in Africa (*n* = 113 samples). The resulting profiles were essentially identical, though with somewhat narrower confidence intervals (electronic supplementary material, figure S1). Consequently, for maximum comparability, the results presented are for the sample size of 85 that could be achieved for all populations.

Each BEAST analysis yields a profile comprising 85 paired size–time estimates that together describe the demographic history of that population. Unfortunately, different populations have different history lengths and the densities of the points vary along each profile. To attempt to obtain a fair estimate of similarity between any given pair of profiles, we used the following strategy. Comparisons were made based on 20 evenly spaced time intervals summing to the length of the shorter history (i.e. 0, *L*/20, 2*L*/20 … *L*, where *L* is the maximum age-point of the population with the shorter history). At each of these 21 time-points, the size of each population was estimated using linear interpolation between the two immediately flanking values, and the total difference was calculated as

$$\lambda =\overset{i=0}{\underset{i=20}{\sum }}|\log(s1)-\log(s2)|,$$

where *s*1 and *s*2 are the interpolated sizes in the two populations at bin *i*.

## 3. Results

The effective sample size (ESS) of relevant parameters was greater than 200, our criterion for convergence, for 19 populations. One South Asian population (BEB) failed to reach 200, so the results for this population should be treated with some caution. However, because replicate subsets all yield similar profiles and the average profile is similar to others from the same geographical region, we believe that the broadly correct demographic history has been recovered. A constant population size can be confidently rejected for all 20 profiles as the 95% highest posterior density for the number of population changes excludes 0 in every instance.

In terms of population similarity, we used autosomal SNP data from the 1000 Genomes Phase 3 to calculate Fst between all population pairs, using the method of Hudson *et al*. [18]. As expected, the major geographical regions are clearly resolved (electronic supplementary material, figure S2). In addition, we also compared the similarity of the demographic profiles obtained using BEAST. Here again, populations from the major geographical regions tend to form discrete clusters (electronic supplementary material, figure S3). Such clustering is consistent with the idea that populations from the same part of the world tend to have experienced similar influences on when and how much they increased in size.

### 3.1. Regional demographic histories

#### 3.1.1. Africa

**Figure 1.**
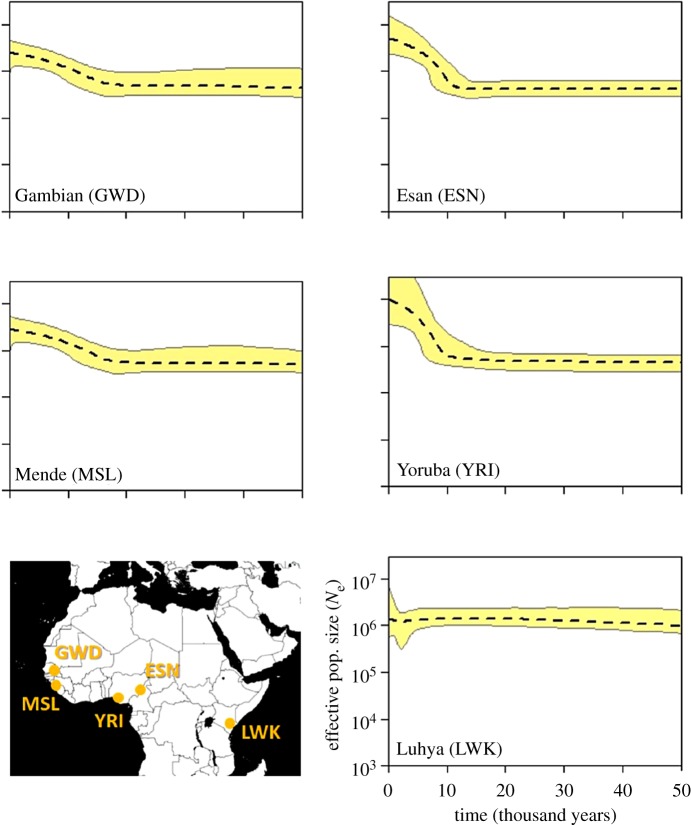
Extended Bayesian skyline plots (EBSPs) for five Africa populations. Each separate population history is inferred from 85 full mitochondrial genomes. Dotted line is the median estimate of effective population size (*N*_e_) and the thin grey lines show the boundary of the 95% central posterior density (CPD) intervals. The *x*-axis represents time from the present in thousands of years. All plots are on the same scale. Map labelled with geographical origins of sampled populations.

Profiles for the five African populations are presented in figure 1. As with all other regions, graphs are arranged to correspond approximately to their geographical locations. All African populations share a large, stable ancestral size that shows little change in the east (Luhya) and an expansion in the west. The signal of expansion is stronger and starts later (around 10–11 ka) in the Nigerian populations Esan and Yoruba compared to the Mende and Gambian populations whose expansion initiates closer to 18 ka. As such, the four West African populations, particularly the Nigerians, echo the profiles found in Southern European populations (see below), albeit with a significantly larger initial size.

#### 3.1.2. Europe

**Figure 2.**
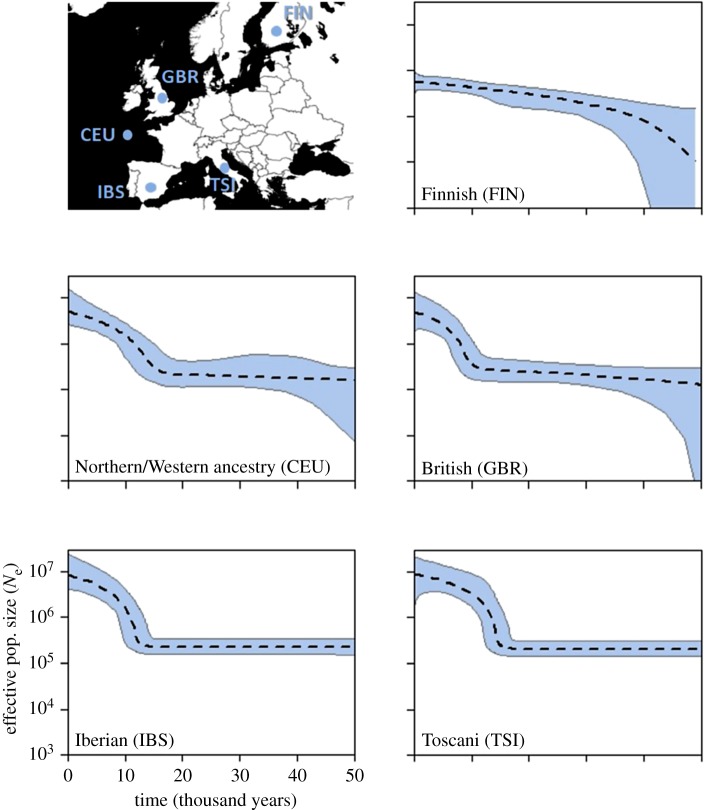
Inferred demographic histories of five European populations. Dotted line is the median estimate of *N*_e_ and the thin grey lines show the boundary of the 95% CPD interval. The *x*-axis represents time from the present in years and all plots are on the same scale. Map shows origins of sampled populations.

The five European profiles are presented in figure 2. The four southerly populations all show profiles with a stable size up to approximately 14 ka followed by a sudden, rapid increase that becomes progressively less steep towards the present. There is also a north-south trend, with confidence intervals becoming broader towards the north, particularly for the oldest time-points. The Finnish population profile appears rather different, but this is to be expected both because it is so far north and because previous studies have identified Finns as a strong genetic outlier in Europe [19–22].

#### 3.1.3. South Asia

**Figure 3.**
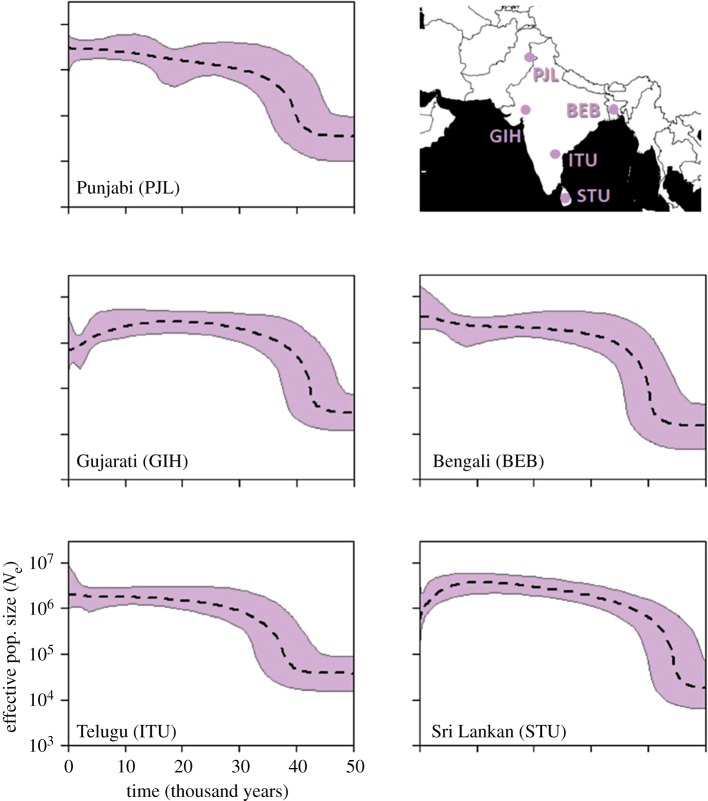
Inferred South Asian population demographic histories. Dotted line is the median *N*_e_ estimate and the thin grey lines show the boundary of the 95% CPD intervals. The *x*-axis represents time from the present in thousands of years and all plots are on the same scale. The map shows location of sampled populations.

The five profiles for South Asia are shown in figure 3. All populations reveal a period of rapid growth approximately 45–40 ka which then slows. Near the present the two southerly populations, GIH and STU both show evidence of a decline. However, this may be due to these samples being drawn from populations no longer living on the subcontinent, with the downward trend capturing a bottleneck associated with moving to Europe/America, perhaps accentuated by the tendency for immigrant populations to group by region, religion and race [23].

#### 3.1.4. East Asia

**Figure 4.**
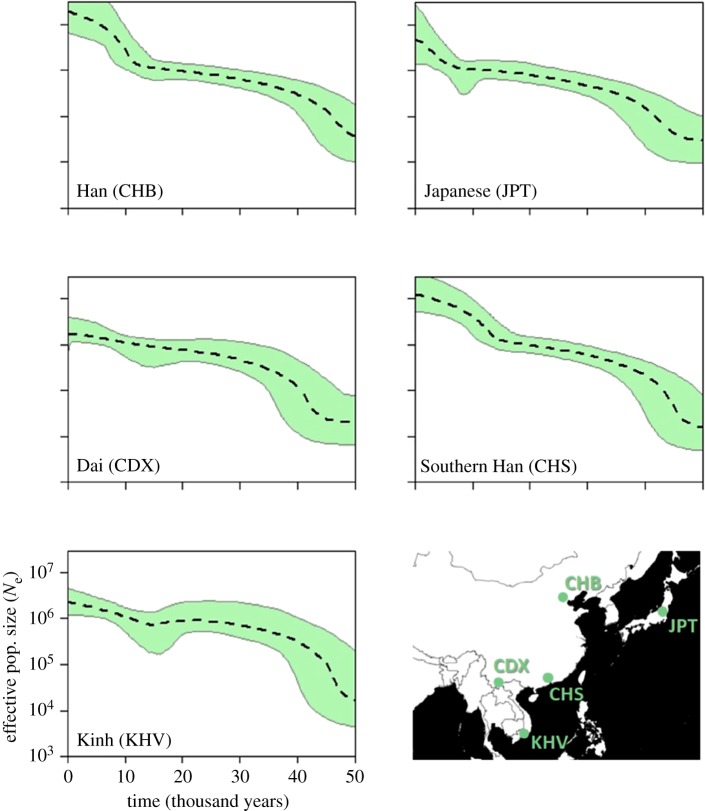
Individual EBSPs of the five East Asian populations. Dotted line is the median estimate of *N*_e_ and the thin grey lines show the boundary of the 95% CPD intervals. The *x*-axis represents time from the present in thousands of years. All plots are on the same scale. Map shows populations sampled.

The five population profiles for East Asia are presented in figure 4. All five profiles show a generally upward trend with variable confidence limits suggesting unresolved demographic complexity. The two south-eastern populations, Dai and Kinh, share similarities with the South Asia group, having a rather rapid increase around 45 ka. The other three populations show a weaker initial expansion, but instead show some similarity to the European populations in terms of a recent accelerated expansion before or around 10 ka. This secondary expansion appears to begin a little later in Japan, as observed by Zheng *et al*. [2].

**Figure 5.**
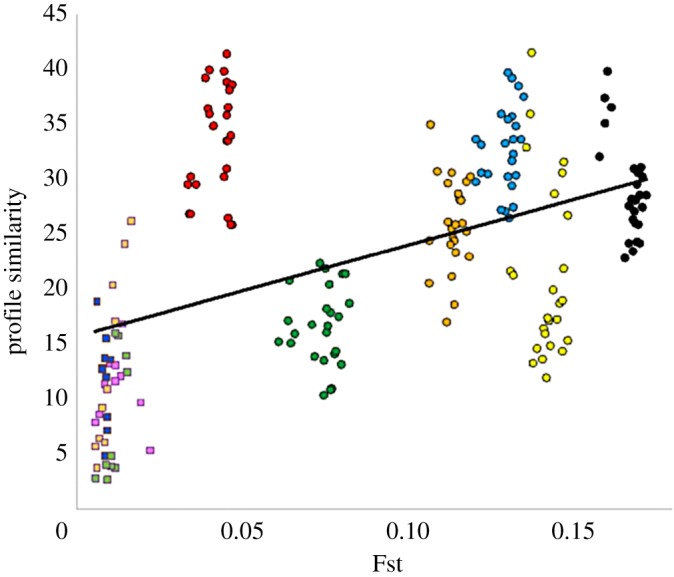
Relationship between profile similarity and genetic distance, measured as Fst. Comparisons between regions, circles, are colour-coded: black = AFR-EA; yellow = AFR-EUR; blue = AFR-SA; orange = EUR-EA; green = EA-SA; red = EUR-SA. Comparisons within regions, squares, are coded: peach = EUR; pink = EA; dark blue = EA; light blue = AFR. Profile similarity is calculated as inferred size difference summed over 20 evenly spaced intervals (see Material and methods).

For a more objective depiction of the extent to which profiles are more similar between related populations, we plotted a measure of curve similarity (CS) against Fst (figure 5). As CS captures differences in both size and profile shape, it is not surprising that the values we find are highly variable. Nonetheless, curve similarity does increase with Fst, and CS values tend to be more similar to each other for particular region–region comparisons compared with the overall range. For example, South Asian profiles seem to have relatively less affinity to Europe and Africa yet greater affinity to East Asia.

## 4. Discussion

We used the Bayesian program BEAST to infer population histories for 20 global human populations using whole mitochondrial genome sequence data from Phase 3 of the 1000 Genomes Project. Our analysis builds on earlier studies using the Phase 1 data e.g. [2,3] or single haplogroups e.g. [22,24]. The Phase 1 data lack any South Asian populations and include several American samples with complex patterns of European admixture [13,14]. By moving to Phase 3 data, we have been able to increase greatly the number of within-region comparisons. We show that populations from the same region show greater similarity between their demographic profiles than populations from different regions. There is also a tendency within each region for the profiles to exhibit geographical trends. While we were able to detect changes in population sizes in all 20 populations, all these increases appear to be too old to represent the effect of the Neolithic transition, in line with previous analyses of more limited datasets [2–5,24]. See electronic supplementary material, table S2.

Compared with previous studies, our analysis has been able to deploy larger sample sizes, a coalescent model and improved mutation rate estimates. The fact that we still fail to detect a clear signal from the Neolithic transition may suggest that even complete mtDNA genomes lack sufficient resolution to detect changes over this time scale. This conclusion agrees with simulations by Aimé & Austerlitz [25], who argued that only microsatellites, which evolve appreciably faster, might offer sufficient genetic resolution to detect such a recent event. Having said this, there may be factors other than sheer mutation rate that confound our ability to detect recent trends. For example, the mitochondrial genome is only a single marker and hence, by chance, may fail to capture signals seen in gene trees produced from other markers. Thus, Silva *et al.* [24] analysed population samples from South Asia, combining autosomal and Y-chromosome markers to reveal patterns consistent with sex-biased dispersal [24]. Here, the lack of a signal of population expansion in mtDNA reflected demographic changes associated with males rather than insufficient mitochondrial mutations.

The possibility that different markers can tell different stories is emphasized by the work of Karmin *et al.* [26]. Their results for mtDNA data are broadly similar to ours, with populations in Africa showing gradual increase over time, an early expansion in Asia and more recent expansion in Europe. However, they use Y-chromosome markers to detect a population reduction in the mid-Holocene, a trend that we fail to detect. One possibility is that the prevailing population structure resulted in relatively stable female effective population size at a time when sex-specific drivers acted to reduce the male *N*_e_.

Verifying the ability of programs like BEAST to infer accurate population histories by simulation is difficult. Modern human populations have extremely complicated histories with changing levels of substructure, stratification by religion and politics and mixing through trade, wars and slavery [27–29]. Yet, at the same time, some level of constancy is maintained through the persistence of insular minority groups. Such complexity seems too great to be captured convincingly by simulations. Consequently, one of the best ways to show success of the method is through the consistency of profiles obtained from independent samples collected from related but distinct populations. The fact that we find profiles that are more similar to each other within a region but differ between regions therefore gives us confidence that we are picking up genuine regional differences: populations that are nearer geographically are more similar in terms of their inferred demographic history, captured more objectively in the general positive trend between Fst and profile similarity. In turn, this pattern also indicates that our sample size of 85 individuals is adequate data for accurate population reconstruction, something we further confirmed by extensive re-running with different, randomly selected subsets.

The reconstructed population profiles we have generated exhibit several features that appear consistent with known demographic events. Thus, the very early expansion observed in East and West Asian populations is compatible with the out-of-Africa bottleneck and subsequent expansion. Similarly, the timing of the expansion in Southern Europe could be seen as pointing to the beginning of the Neolithic Transition in the Near East, the source of farmers who later colonized the rest of Europe [30–32]. In both these cases, the expansion signals reflect older events that probably happened before the lineages arrived at where they were sampled. Equally, the profiles and expansion dates we find across South Asia are similar to those found in previous studies [24,33] such as work by Silva *et al*. who suggest that the expansion signal seen in their BSPs around 45–35 ka may be indicative of a secondary founder event in the region that obliterated more ancient signals.

Within each major region, the profiles are generally rather similar, though interestingly there also appear to be east–west/north–south trends. Thus, in Africa, the two westernmost populations GWD and MSL both show an earlier but smaller expansion compared with the two Nigerian populations YRI and ESN. Similarly, among the East Asian populations, there is tendency for the most recent expansion to occur more recently in the more northern populations CDX, CHB and JPT. It is also notable that the more northern/eastern South Asian populations have profiles that are most similar to the more western East Asian populations, with PJL and ITU appearing most similar to CDX and KHV. These putative trends require a further increase in sample size to quantify but suggest that demographic change can, in principle, be tracked across both time and space.

The fact that the earliest signals are found in populations that are mostly far from where they were when the changes occurred raises an important cautionary note in interpreting these trajectories: such reconstructions are only valid under the assumption of a closed population [10,34,35]. Population structure, expansions and mixing all generate apparent changes in *N*_e_ which might have nothing to do with actual changes in the local census population. A single uniparental marker offers a powerful tool for investigating demographic histories, but interpretation must be done carefully with the understanding of what details might be missing, wiped out or swamped by a suite of different influential processes [24]. This issue is not specific to BEAST, and other approaches such as PSMC (pairwise sequentially Markovian coalescent) suffer of the same limitations [36]. It is this need to avoid likely admixed populations that caused us to exclude populations from the Americas.

In conclusion, expansion of the analysis of the 1000 Genomes Project mitochondrial DNA data to Phase 3 allows novel comparisons both between and within four major geographical regions. Although it remains difficult to ground-truth the dates, the fact that clear geographical trends are apparent suggests that the relative size and timing of expansions found are probably reliable. However, naive interpretation of the data would imply that the populations studied all experienced expansions that initiated prior to the adoption of agriculture. It was previously suggested that such changes might be associated with changes in lifestyle, such as an increase in sedentarism, that occurred before the advent of food production in the Neolithic [1]. Rather, we suggest that the signal from each local population in fact reflects a much deeper demographic history, not from those local derived populations being studied, but older ‘source’ populations which underwent geographical expansions.

## Supplementary Material

Supplementary Figure 1. EBSPs of populations with the largest sample sizes from each of the four major regions.

## Supplementary Material

Supplementary Figure 2. Neighbour-joining tree based on Fst from autosomal SNPs.

## Supplementary Material

Supplementary Figure 3. Neighbour-joining tree based on the similarity of EBSP profiles.

## Supplementary Material

Supplementary Figure 4. Global position of sampling sites with all profiles from each major region overlaid.

## Supplementary Material

Supplementary Table 1. Partitioning scheme for mtDNA.

## Supplementary Material

Supplementary Table 2. Summary table of values for population N_e_ and key dates.

## Data accessibility

Genetic sequence data used in this study were downloaded from the 1000 Genomes Project website—www.1000genomes.org/data.
