# Supplementary figures and images for: Global demographic history of human populations inferred from whole mitochondrial genomes
Source: R Soc Open Sci. 2018 Aug 22;5(8):180543. doi: 10.1098/rsos.180543 (PMC6124094; doi:10.1098/rsos.180543)

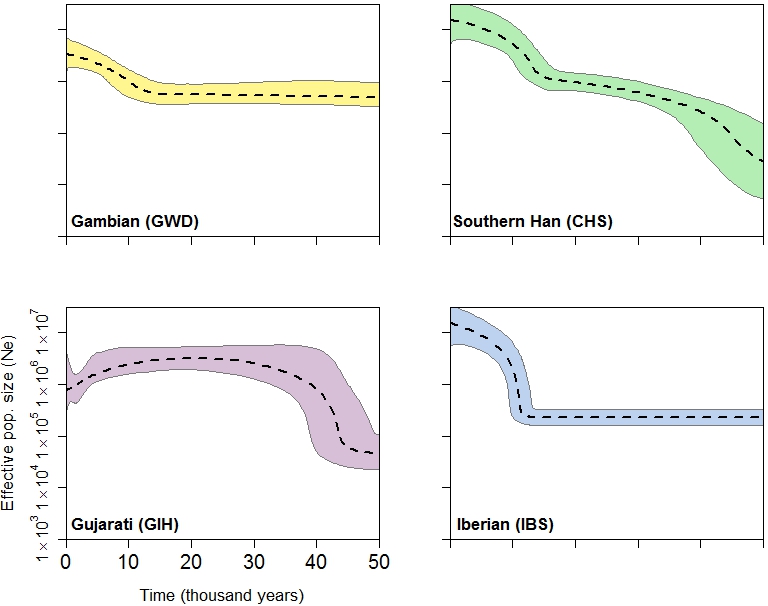

Supplement: Supplementary Figure 1. EBSPs of populations with the largest sample sizes from each of the four major regions. [file rsos180543supp1.jpeg]

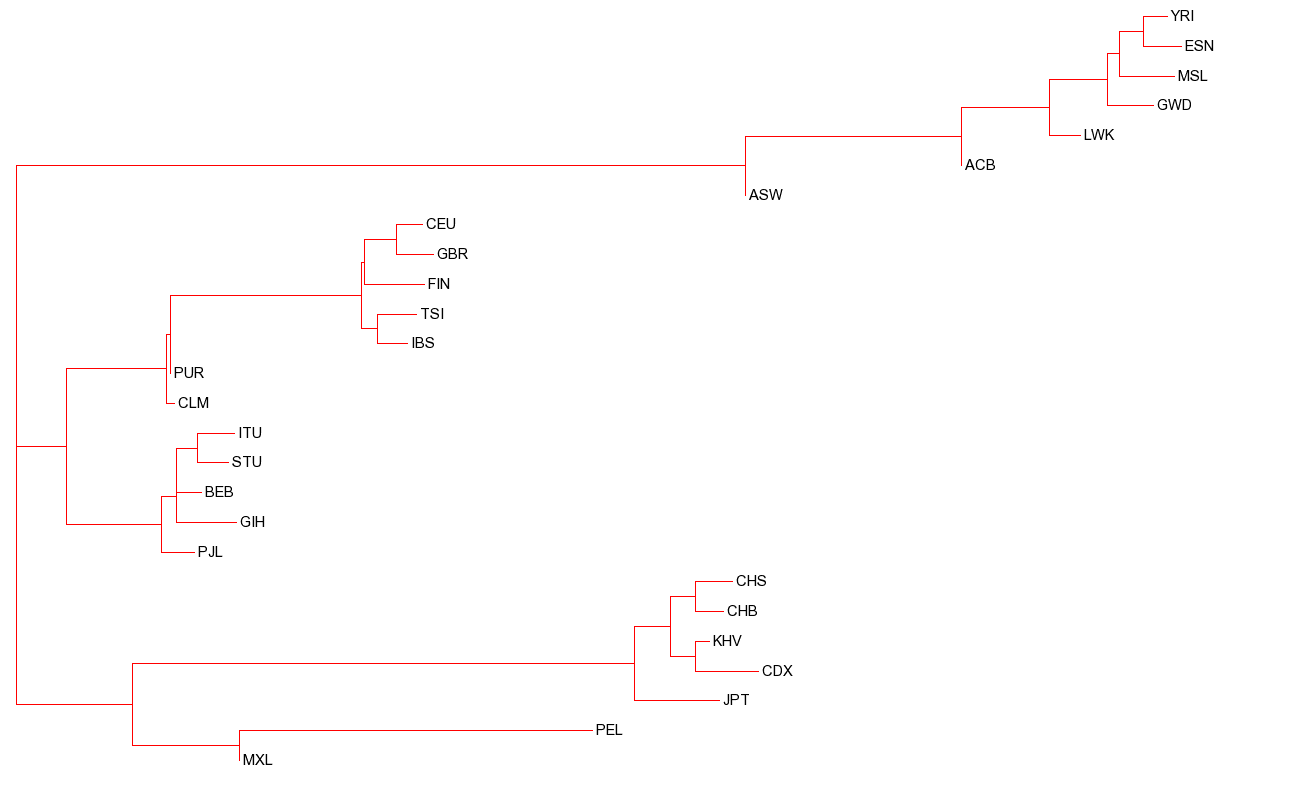

Supplement: Supplementary Figure 2. Neighbour-joining tree based on Fst from autosomal SNPs. [file rsos180543supp2.png]

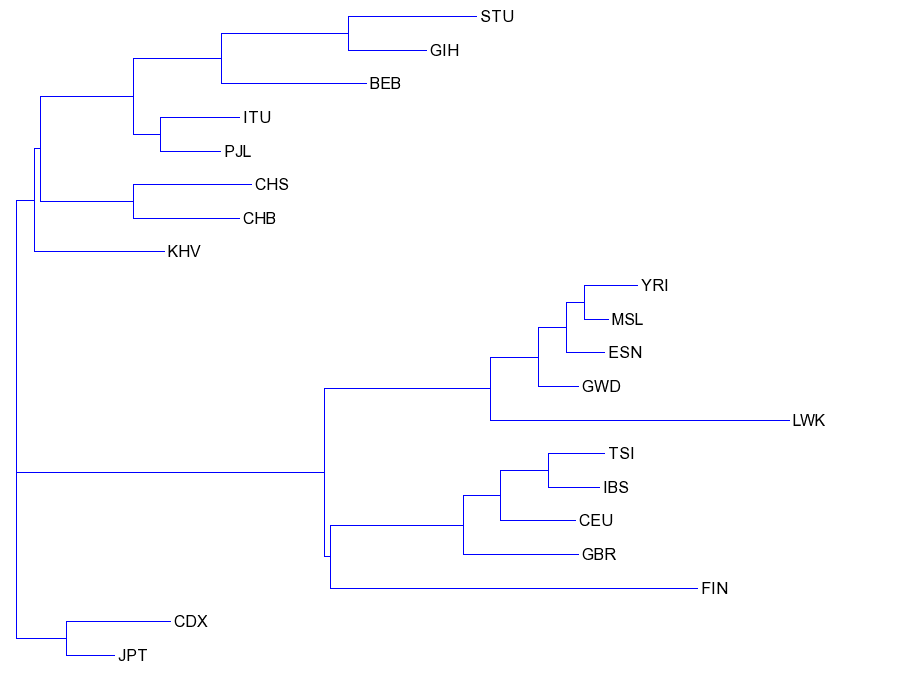

Supplement: Supplementary Figure 3. Neighbour-joining tree based on the similarity of EBSP profiles. [file rsos180543supp3.png]

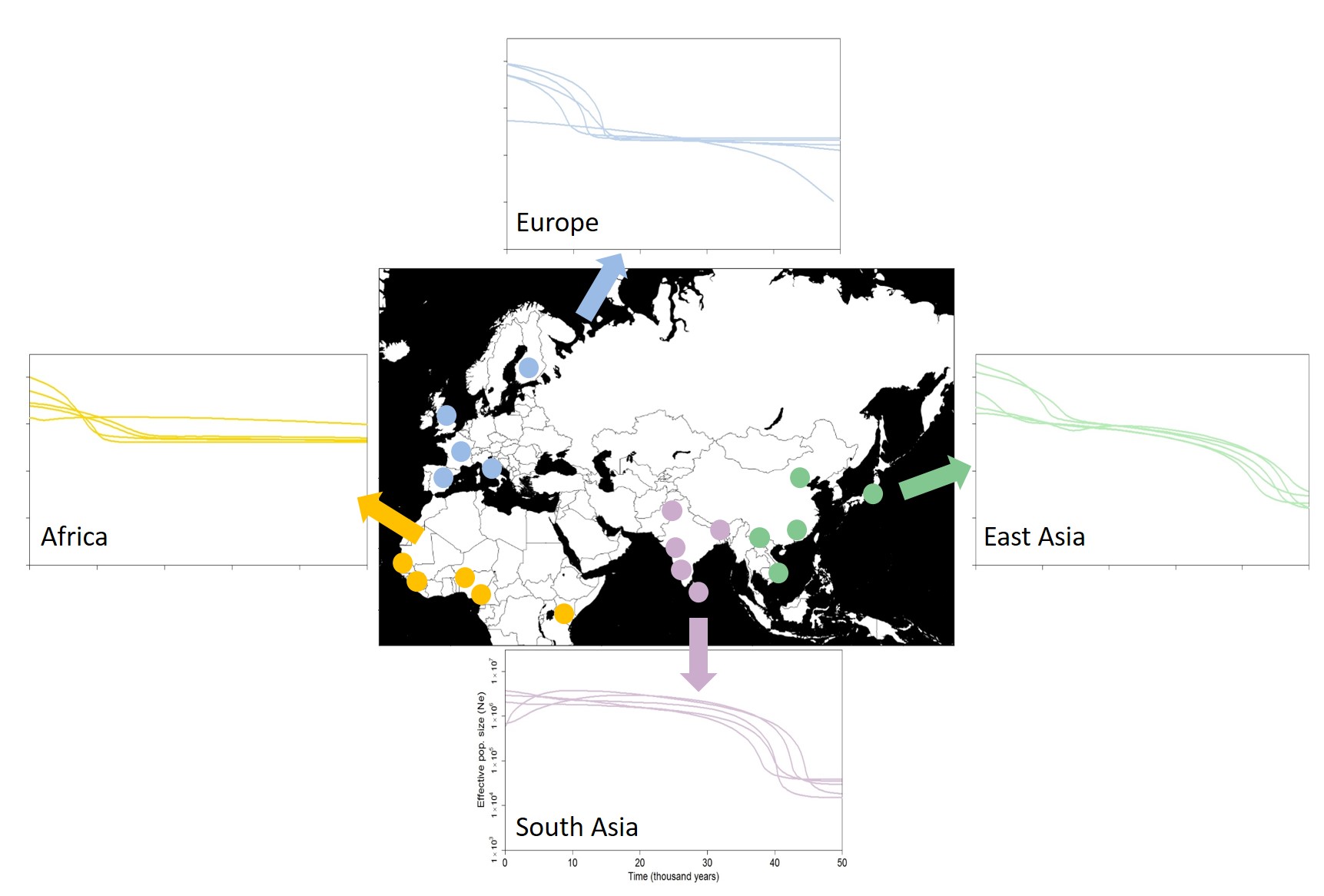

Supplement: Supplementary Figure 4. Global position of sampling sites with all profiles from each major region overlaid. [file rsos180543supp4.jpg]

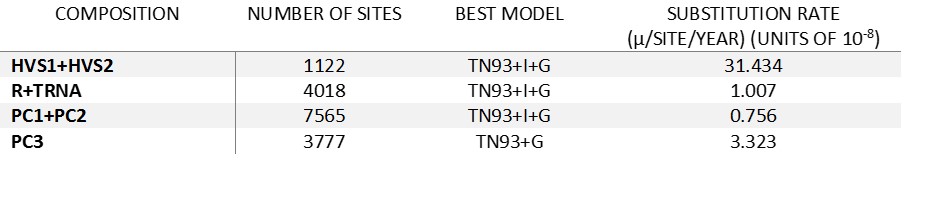

Supplement: Supplementary Table 1. Partitioning scheme for mtDNA. [file rsos180543supp5.jpg]

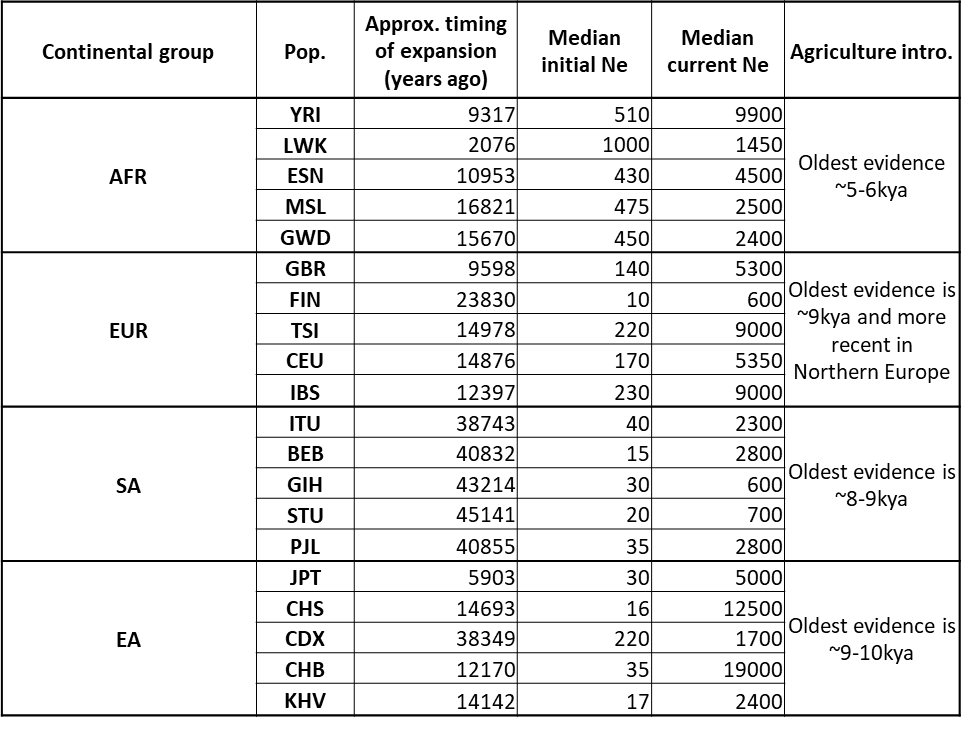

Supplement: Supplementary Table 2. Summary table of values for population Ne and key dates. [file rsos180543supp6.png]
